# Unicystic ameloblastoma appeared as a massive multilocular entity: A case report with long-term follow-up

**DOI:** 10.1016/j.ijscr.2024.109830

**Published:** 2024-05-29

**Authors:** Mhammad Ali, Alaq Qassem, Sara Tawashi, Karam Ahmad, Abdul-Karim Khalil

**Affiliations:** a Al-Andalus University for Medical Science, Faculty of Dentistry, Syria; b Al-Wataniya Private University, Faculty of Dentistry, Syria; c Tishreen University Hospital, Department of Oral and Maxillofacial Surgery, Syria; d Al-Andalus University for Medical Sciences, Department of Oral and Maxillofacial Surgery, Syria

**Keywords:** Unicystic ameloblastoma, Odontogenic tumors, Oral and maxillofacial surgery, Case report

## Abstract

**Introduction:**

Unicystic ameloblastomas are a rare variant of ameloblastomas, which are characterized by slow growth and being relatively locally aggressive, with the main site of origin being the posterior portion of the mandible, it also refers to those cystic lesions that show clinical, radiographic, or gross features of a jaw cyst.

**Presentation of case:**

A 27-year-old female patient presented with a chief complaint of extensive mass of mandible along with severe swelling and numbness of right lips and chin. The oral examination revealed a swelling in the molar region of the right mandible with buccal plate expansion. The radiographic and histopathologic features were consistent with the diagnosis of unicystic ameloblastoma. Consequently, the lesion was surgically removed, and no clinical or radiological recurrence was detected during 5 years post-operative follow-up.

**Discussion:**

While previous reports of unicystic ameloblastoma in the posterior portion of the jaw showed favorable prognosis lesions appeared as a unilocular entity, this case reports multilocular appearance and aggressive behavior of expansive unicystic ameloblastoma. Furthermore, while some studies linked the unilocular appearance of unicystic ameloblastoma to impacted tooth, our case suggests a possible traumatic link of preexisting lesion into multilocular unicystic ameloblastoma related to impacted tooth.

**Conclusions:**

This case presents a rare multilocular unicystic ameloblastoma appearance, notably with impacted tooth involvement. It also indicates the potential transformation of solid ameloblastoma into unicystic ameloblastom.

## Highlights

• This case represents distinctive features of Unicystic Ameloblastoma with abnormal behavior.
• This UA case suggests the potential transformation of preexisting Solid Ameloblastoma into Unicystic Ameloblastoma.
• This case pays attention to the importance of long-term follow-up in UA mural subtype cases.

## 1 Introduction

Ameloblastoma refers to a benign odontogenic tumor of epithelial origin and is locally aggressive with unlimited growth capacity [1,2], It is typically classified as unicystic, multicystic, peripheral, and malignant subtypes [3]. The lesion has a potential to present aggressively, it occurs primarily in middle-aged adults, with a predilection for the posterior mandible. Radiographically, it exhibits an expansile unilocular or, more often, multilocular pattern with discrete margins, and association with an impacted tooth is commonly observed [2].

Unicystic ameloblastomas are a rare variant of ameloblastomas, which usually occur in younger populations. They are characterized by slow growth and being relatively locally aggressive, with the main site of origin being the posterior portion of the mandible. Late recurrence following surgical management is relatively common and is related to the histological type, the site of origin, and the initial treatment modality [4].

The term unicystic ameloblastoma refers to those cystic lesions that show clinical, radiographic, or gross features of a jaw cyst, but on histologic examination show a typical ameloblastomatous epithelium lining part of the cyst cavity, with or without luminal and/or mural tumor growth [5]. The unicystic variant of ameloblastoma is generally considered less aggressive than its solid or multicystic forms and is typically treated with enucleation or curettage. However, if it is of the unicystic ameloblastoma mural subtype, treatment by marginal resection is recommended [5]. Recurrence of unicystic ameloblastoma may be long delayed, and a long-term postoperative follow up is essential to the proper management of these patients [5]. This case represents a rare manifestation of unicystic ameloblastoma and pay attention to the potential transformation of preexisting lesion into unicystic ameloblastoma. The work has been reported in line with the SCARE 2023 criteria [6].

## 2 Presentation of case

A 27-year-old female patient was referred to the department of oral and maxillofacial surgery in February 2019 with a chief complaint of huge mass on the right side of mandible that appeared externally along with severe swelling and numbness of right lips and chin. The patient reported that it was first appeared spontaneously 14 months ago, and gradually increased in size resulted in teeth misalignment and chewing difficulty. No significant or serious events neither symptoms was reported in the patient's medical history and her oral hygiene showed no additional abnormalities generally. Overall, the patient has no history of chronic illnesses, surgeries, or hospitalizations, and she has a healthy lifestyle and no significant health concerns.

The extraoral examination revealed a diffuse swelling over the body of mandible and mandibular angle, and buccal expansion on the overlying skin caused facial asymmetry, which was obviously noticed. The swelling was hard in palpation and the overlying cortex was thinned out. Intraoral features showed clearly the expansion of buccal and lingual cortices from the tooth 45 extending to the retromolar triangle area and the anterior border of ascending ramus, the teeth 44, 45, 46, 47 were in malposition whether the tooth 48 was not visible. Diffuse swelling in the buccal and lingual vestibule appeared around the posterior portion of mandible with no ulceration or drainage related.

**Fig. 1:**
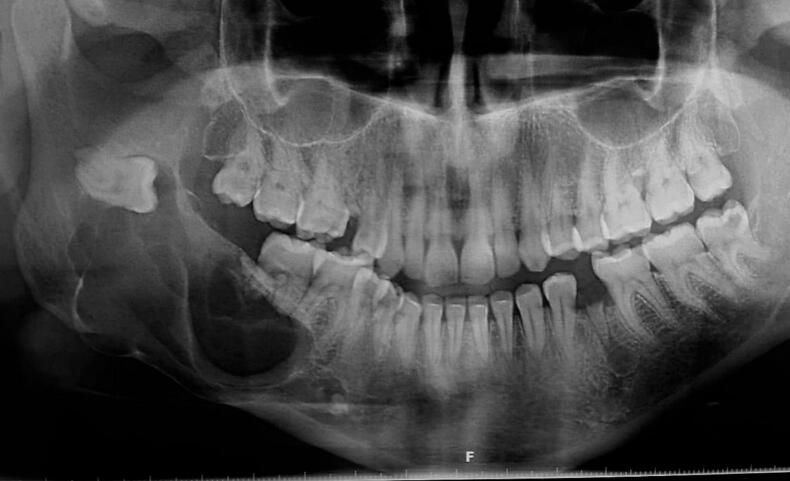
OPG revealed a huge multilocular appearance involved the main body of mandible and ascending ramus.

OPG displayed an extensive well-defined macro-multilocular mass of the main body of mandible measuring approximately 3 × 5 cm extending to the ascending ramus and related to the crown of impacted tooth 48. Radiographic features showed extensive osteodestruction and thin corticated borders with outspread expansion of the buccal side of mandible. Root resorption on the tooth 47 and distal root of tooth 46 was observed with intensive displacement of the adjacent teeth of the lesion, and all the teeth related to the lesion were vital including the impacted tooth 48 [Fig. 1]. Odontogenic keratocyst, central giant cell granuloma and solid ameloblastoma were considered for the differential diagnosis.

**Fig. 2:**
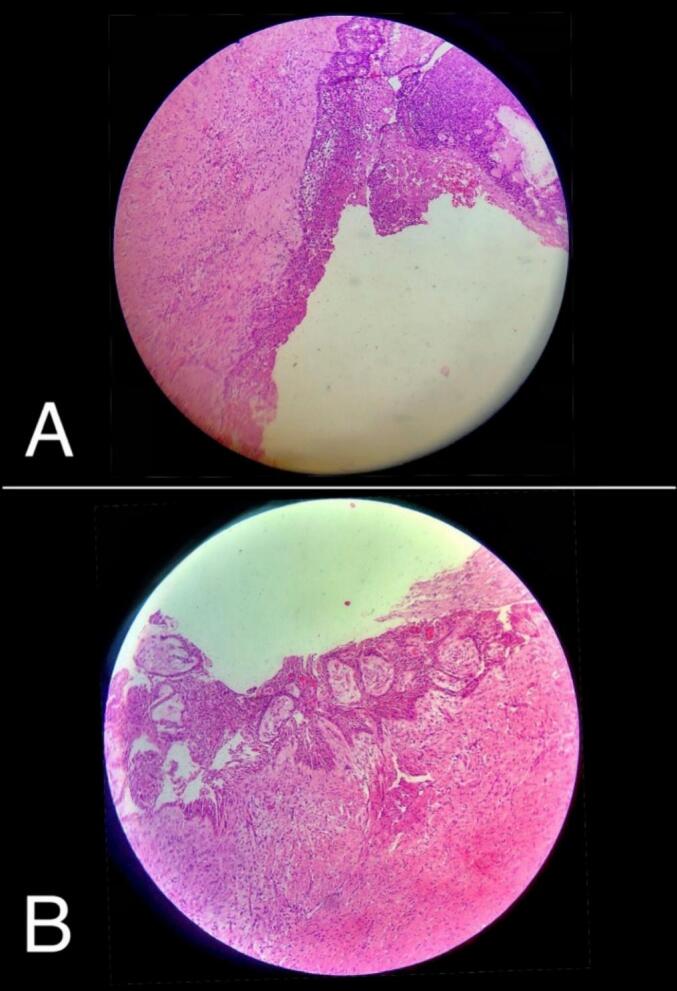
(A): The epithelial lining of unicystic ameloblastoma with intraluminal proliferation, (B): Intramural proliferation of the epithelial lining cyst.

An incisional biopsy procedure was performed, and two pieces of fragments measuring 1 × 0.8 cm that appeared as tan-firm tissues covered by mucosa cut sections were sent to the histopathological department. Microscopical examination revealed cystic capsule covered by ameloblastic epithelium with proliferation strands of neoplastic epithelial cells involved the connective tissue wall and presenting a predominantly plexiform pattern, the suprabasal layer showed intraluminal satellate-reticulum proliferation with no vaculation [Fig. 2], and these features were consistent with the diagnosis of ameloblastoma, unicystic subtype.

**Fig. 3:**
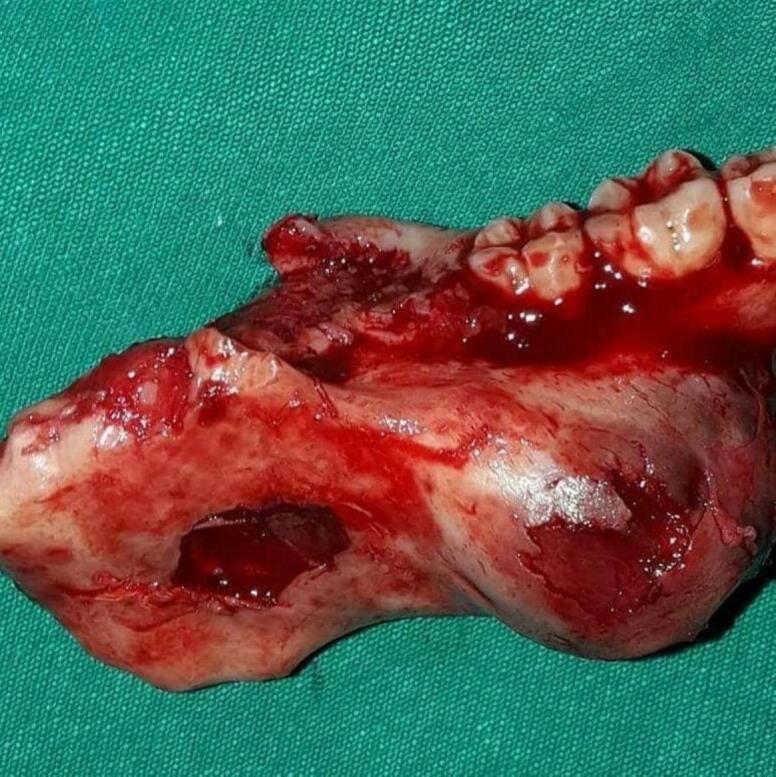
Intraoperative figure of the lesion after segmental resection en-bloc.

**Fig. 4:**
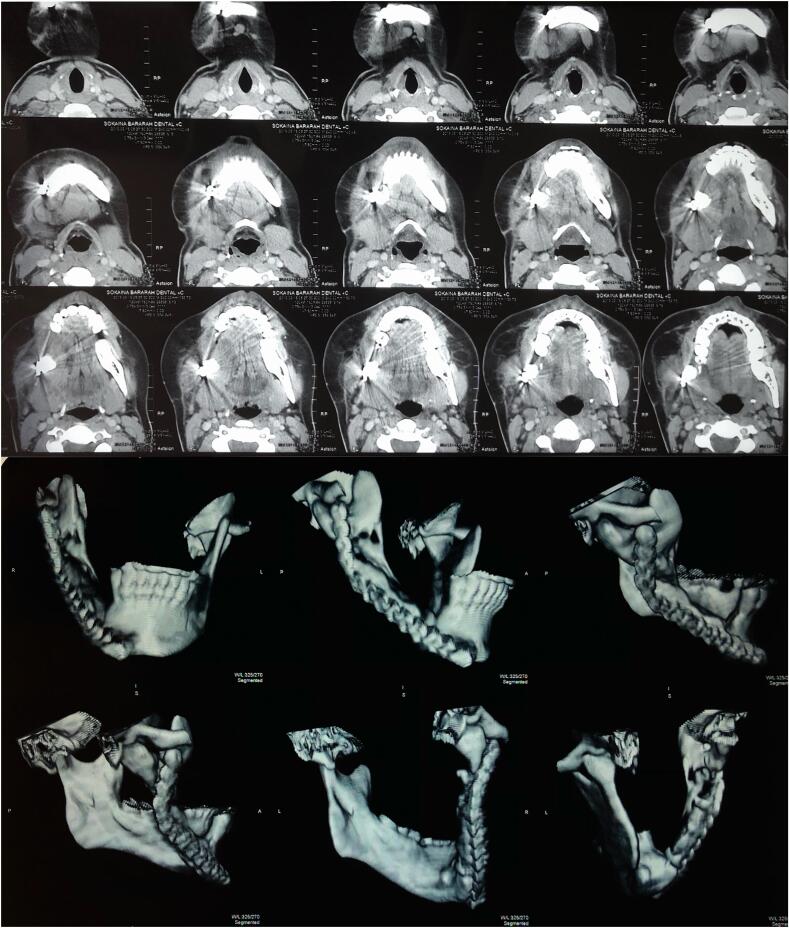
Post-operative CT and CBCT shows reconstruction plate in the region of excision.

Depending on clinical and radiographic findings of the case we presented, surgical intervention using segmental resection was applied as a treatment procedure to remove the lesion due to the aggressive behavior and destructive manifestations related that renders conservative management contraindicated. The lesion was enucleated en-bloc with bony security margin of 1 cm extending into the mandibular ramus to the tooth 44; the teeth 45, 46, 47, and 48 were involved [Fig. 3]. We used reconstruction plate in the region of excision considering patient's perspective and the aim of achieving the functional and aesthetic recovery in order to improve the patient's postoperative quality of life [Fig. 4].

**Fig. 5:**
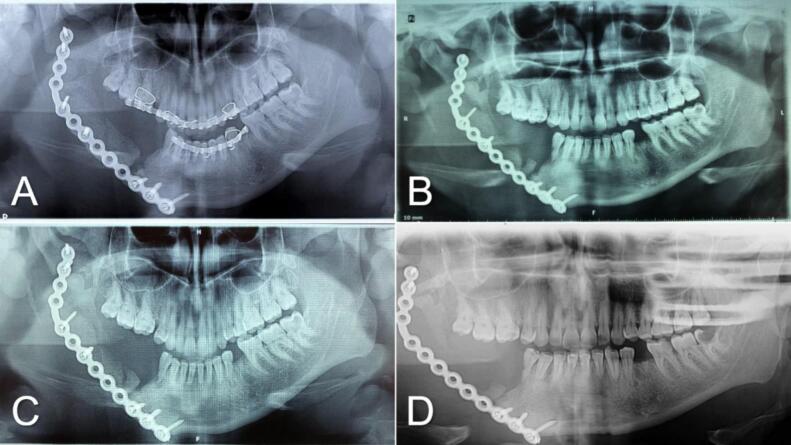
OPGs of post-operative follow-up (A): After 3 months, (B): After 1 year, (C): After 3 years, (D): After 5 years.

The follow-up over the first 5 years after surgery was considered to monitor the patient's progress and assess the efficacy of the surgical intervention. However, the follow-up was eventless without any signs of recurrence or post-operative complications [Fig. 5].

## 3 Discussion

This case reports a rare clinical behavior and radiographic appearance of unicystic ameloblastoma affecting the right mandibular angle, associated with mild jaw pain, swelling and numbness as main clinical symptoms. Unicystic ameloblastoma is a less encountered variant of the ameloblastoma, referring to cystic lesions that appear frequently in second to third decade of life [5]. It pretends to be asymptomatic until develops into gross expansion that makes it more deleterious, and one of the most typical features of unicystic ameloblastoma is the ability to mimic an odontogenic cyst radiologically and clinically. The case we presented is a type of enlarged unicystic ameloblastoma in 27-year-old female patient that appeared spontaneously with no significant symptoms or surgical procedures related to her medical history and left untreated from the first time noticed 14 months previously.

Radiographically, unicystic ameloblastoma commonly shows expansive unilocular radiolucency with well-defined borders, the unilocular pattern is more common in the unicystic variant than the multilocular one, and there is a clear predominance of a unilocular configuration in all studies of unicystic ameloblastoma where this feature has been evaluated, especially in cases associated with tooth impaction [2,7]. Despite, our case represents a pericoronal-scalloped radiolucency on panoramic radiograph that appeared as a macro-multilocular mass associated with impacted third molar, wide expansion and cortical perforation on the buccal side were also noticed. Notably, it is unusual to see cortical perforations or bone erosions related to unicystic ameloblastoma cases [2].

Various studies described the development of unicystic ameloblastomas, and many contradictory theories have been proposed. Some authors suggest that unicystic ameloblastoma may develop by mural and/or luminal ameloblastomous changes in preexisting cyst [8]. Robert and Diane have determined three pathogenic mechanisms for the evolution of unicystic ameloblastoma, which could be secondary to reduced enamel epithelium, previous localized dentigerous cyst or due to cystic degeneration of solid ameloblastoma [9]. In the present case, the impacted tooth, macro-multilocular appearance, and unicystic variant of the mural type, which presents as a plexiform pattern, support the potential of third mechanism. This mechanism involves cystic degeneration of ameloblastic islands in a solid ameloblastoma followed by the fusion of multiple microcysts, resulting in a unicystic ameloblastoma.

The treatment modalities of the represented type of ameloblastoma remains controversial because it is a benign, locally aggressive tumor with a high recurrence rate. The histopathological classification should be mainly concerned in case of management unicystic ameloblastoma, whereas the intramural proliferation subtype has a high recurrence rate and indicates to be treated by segmental resection as for a solid or multicystic ameloblastoma [10]. In our case, the histopathological findings illustrated the luminal and intramural growth, which consist with unicystic ameloblastoma. Additionally, the radiographic features showed wide destruction of the lower borders of mandible extending to the ascending ramus, which is more dangerous and leads to high complications in bone regeneration process if treated conservatively by enucleation or curettage [11]. Based on histopathology, there are various treatment options for cystic ameloblastoma, such as enucleation. However, we have opted for resection. Accordingly, the surgical procedure using segmental resection was performed as a treatment method and long-term follow-up over the last 5 years post-operatively was mandatory based on the increased relapse cases within 5 years after operation, and to avoid underestimation of the recurrence rate due to the abnormal behavior that represented in this case [5,12].

## 4 Conclusions

This case highlights a rare occurrence of pericoronal unicystic ameloblastoma associated with an impacted third molar. The lesion presented as a macro-multilocular mass, suggesting the potential transformation of a preexisting solid ameloblastoma into a unicystic form. Furthermore, it underscores the significance of long-term follow-up in cases presenting with abnormal behavior.

## Informed consent

Written informed consent was obtained from the patient for publication of this case report and accompanying images. A copy of the written consent is available for review by the Editor-in-Chief of this journal on request.

## Ethical approval

Ethical approval is waived at our institution and this study was exempt from ethical approval at our institution, as this paper reports a single case that emerged during normal surgical case report.

## Funding

No source of funding.

## Author contribution

MA: Writing and drafting the article, Critical Revision.

AQ, ST: Data Collection, preparation and visualization.

AKh, KA: Supervision.

All authors provided final approval of the version to be submitted.

## Guarantor

Abdul-Karim Khalil DDS, OMFS, PhD.

## Research registration number

N/A.

## Conflict of interest statement

None.
